# Factors associated with antiretroviral therapy adherence among people living with HIV in Haiti: a cross-sectional study

**DOI:** 10.1186/s12981-021-00405-4

**Published:** 2021-11-02

**Authors:** Ludentz Dorcélus, Joseph Bernard, Constant Georgery, Clerveau Vanessa

**Affiliations:** 1 Zanmi Lasante, Hôpital Saint-Nicolas, Saint-Marc, Haïti; 2 Université Notre-Dame d’Haïti, Rue Sapotille #4, Port-au-Prince, Haïti; 3 Zanmi Lasante, Santo 18, Croix-des-Bouquets, Haïti

**Keywords:** Adherence HIV, Haiti, Antiretroviral therapy

## Abstract

**Background:**

Socioeconomic, demographic and clinical factors can affect adherence to treatment among people living with HIV (PLH) and potentially have an impact on their prognosis and survival. The main objective of this study was to assess these factors as potential barriers to adherence among patients receiving care in central Haiti.

**Methods:**

A cross-sectional study was conducted among PLH receiving antiretroviral therapy (ART) at the TB/HIV clinic at St. Therese Hospital in Hinche, Haiti. A total of 426 potential participants were approached during their follow-up visits from June to August 2019, of whom 411 participated in the study. After giving informed consent, study participants completed a structured interview that included the Self-Report Item Scale (SRIS), a standard measure, to assess adherence. Socio-demographic, economic and clinical factors were assessed for their association with adherence.

**Results:**

The 411 participating patients represented 39% of the patient population at the TB/HIV clinic during the timeframe of the study. The mean age was 43.7 years (range: 19–80), 65.5% were female and 78.1% had only achieved a primary level of schooling. Nearly 78% had received ART for less than 10 years, 3.41% reported having poor adherence and 28% less than excellent adherence. Factors related to poor adherence in bivariate analysis were age less than 40 years (OR: 6.32, 95% CI 2.04–10.58, p < 0.01) and inability to meet basic needs (OR: 2.70, 95% CI 1.04–7.0, p = 0.03).

**Conclusions:**

To improve medication adherence, the hospital should strengthen patient counselling of younger recipients of ART and provide financial assistance and other social service interventions. Studies should be implemented in other HIV management centers in Haiti and similar contexts to examine barriers to ART adherence with the goal of improving prognosis and survival in the long-term among PLH in resource-limited setting.

## Background

Suboptimal adherence to antiretroviral therapy (ART) is associated with a higher probability of drug resistance and virological failure [1]. Adherence is, therefore, a powerful determinant of the quality of life and survival among people living with HIV (PLH) [2]. The World Health Organization (WHO) defines adherence as the degree to which the patient’s behavior (including medication uptake) matches a health worker’s recommendations. Adherence factors can be related to four dimensions: the patient, the disease, the patient-physician relationship and treatment management [3]. Factors related to the patient include socioeconomic, demographic, psychological, cognitive and behavioral variables [4]. Hence, employment, income, and education can play an important role in the patient’s understanding of his/her illness and ability to participate in his/her management.

According to previous studies, there is a positive relationship between higher levels of education and better living conditions with ART adherence. This may be in part related to greater understanding of what treatment entails as well as having the economic capacity to access treatment [5]. While 85% of the participants in a study published by Malow et. al reported perfect adherence, education was not an associated factor [6]. Employed patients were more likely to adhere to ART, as it is associated with better social support, better structuring of time and improved psychological well-being [7]. A meta-analysis of mostly observational studies concluded that employed PLH from low and high income countries were more likely to adhere to ART [8]. Unemployment [9], lower educational level, profession [10], age > 40 years [11], gender female [12] were associated with poor adherence in these studies conducted in Latin America and Brazil. In central Haiti, PLH with low socio-economic status often have to travel extensively to obtain adequate medical care, although ART itself is supplied free of charge. PLH typically receive a regimen with a number of drugs that need to be taken on a daily basis, which can be challenging if he/she has limitations in understanding HIV disease and its management, given that the literacy rate is estimated as 61% [13].

To our knowledge, there were no published studies on factors associated with adherence to ART in the central region of Haiti. There were studies that examined risk factors of delayed viral suppression around the Port-au-Prince area of Haiti [14, 15]. The central region of Haiti is the 5th highest of the 10 departments of the country in terms of population, with 726,326 people counted in 2015. About one-fifth of the population lived in Hinche, the capital of the Central Department [16]. This department showed the lowest prevalence of PLH with 1.2%, while it is 2% country-wide [17]. The PNLS, an institution under the guidance of the Haitian ministry of health proposed, at that time, 3 regimens for ART: 1st line, 2 nucleoside reverse transcriptase inhibitors + 1 non-nucleoside reverse transcriptase inhibitor; 2nd line, 2 nucleoside reverse transcriptase inhibitor, 3rd line, DRV/r + RAL + ETV [18]. The aim of this study was to examine sociodemographic and economic factors associated with adherence to ART in PLH enrolled in the HIV/TB clinic at St. Therese Hospital in Central Haiti.

## Methods

### Aim

The aim of this study was to determine the factors associated with ART adherence in one hospital in the Central Plateau region of Haiti.

### Study design and setting

This cross-sectional study was implemented with PLH receiving ART seen in consultation at St. Therese Hospital, a public facility in central Haiti which is also a site affiliated with Partners In Health/ Zanmi Lasante (PIH/ZL), a non-governmental organization. Data were collected through a structured questionnaire with additional treatment information retrieved from the Electronic Medical Record for the HIV program at the hospital.

### Study population

A total of 426 patients were approached for purposes of recruitment during their scheduled follow-up visit from June 17 to August 14, 2019. Of those, one patient refused to participate; and 425 (99%) agreed to participate. The eligibility criteria included having received ART for 1 month or longer during this time period, being 18 years of age or older and having received ART administered through the clinic at St. Therese hospital. Patients with speech or memory problems and hence could not answer questions about socio-demographic characteristics and patients who had been on ART for less than a month were excluded from participation. The study sample reduced to 411, as 10 recruits were less than 18 years of age, two people had speech/memory problems, and two had received ART for less than a month prior to the start of the study.

### Data collection and statistical analysis

Data were collected using a structured questionnaire after the participants’ routine medical consultation. Participants were interviewed by two trained research assistants. The questionnaire was designed for this study, and variables were chosen based on information found in the literature (Table 1). Descriptive statistics were calculated with frequencies and percentages. Full sociodemographic and clinical characteristics can be found in Tables 2 and 3. A bivariate analysis was conducted to examine the association between ART adherence and the following variables: sex, place of residence, number of years of treatment, time to arrive at the hospital, adherence, level of education, employment, housing, ART regimen and ability to meet basic needs. “Ability to meet basic needs” was based on the participants’ self-perceived ability to meet these needs. In the local context, this is typically related to whether the people could afford basic needs like water/food supply, had access to electricity and school for their children. If the patient lives in the city of Hinche, we would consider them living in urban area. Outside the city would imply living in rural area. Additional data are mentioned in Tables 4 and 5. Level of education was classified based on the studies on our review of literature. It was classified as not-schooled for PLH with no level of education and schooled with PLH with some level education for multivariate analysis. Employment was defined as having a job at the time of interview. It was classified as employed vs. non-employed (no job, sick/disabled, retired) for multivariate analysis. Housing was defined as ownership status of the house they live in, classified as owner vs. non-owner (tenant, living with family and friends) for multivariate analysis. Ability to meet basic needs would be dichotomous, with yes reflecting always, most of the time or sometimes and no indicating never. Adherence was measured with the Self-Report Scale Item which asked the following question: “Pandan mwa ki sot pase a la a, kijan ou panse ou te pran medikaman yo?” which can be translated to “During this past month, how well do you believe you’ve taken your medication?” [19] This item was based on a five-point Likert scale: excellent, very good, good, acceptable, poor, with the variables “acceptable” and “poor” defined as “poor adherence” for this analysis. For multivariate analysis, adherence would be dichotomous, with poor and acceptable adherence classified as bad adherence, and excellent, very good and good adherence classified as good adherence. Associations were calculated using Mantel–Haenszel chi-square statistics, odds ratios, and corresponding 95% confidence intervals. Statistical analysis was performed using the Epi Info 7.2.2.6 software. The literature review that helped us develop the questionnaire can be resumed in Table 1.

**Table 1:** Sources from the literature for our variables

Variables	Sources
Gender	Gender equality (17) Better adherence amongst men (37, 38)
Age	No influence based on age (30) Better adherence amongst patients over 40 (26, 27, 29)
Area of residence	Better adherence amongst patients in rural area (39)
Time taken to get to the hospital	Better adherence amongst patients that live near the hospital (40)
Economic means	Poor adherence (28, 45, 46)
Education level	No influence (22, 31) Better adherence amongst patients with better education level (23, 24) Lost to follow-up amongst non-schooled patients (28)
Employment status	No influence (23, 30, 31)
Workload	Poor adherence (42, 43)
Housing status	Poor adherence amongst home owners (27))
Social interactions	Poor adherence amongst patients living alone (37) Good adherence amongst patients with multiple social activities (44)
ART	Poor adherence amongst patients experiencing side effects to ART (27, 28, 31)

**Table 2:** Sociodemographic characteristics of the study population

Variables	N = 411 (%)	95 CI %
Gender		
Female	269 (65.4)	60.6–70.0
Male	142 (34.5)	30.0–39.4
Age		
< 30	63 (15.3)	12.26–19.06
30–39	91 (22.1)	18.28–26.53
40–49	113 (27.4)	23.29–32.13
50–59	99 (24.0)	20.09–28.58
60–69	33 (9.0)	5.67–11.20
≥ 70	12 (2.9)	1.59–5.18
Area of residence		
Rural	242 (58.8)	53.94–63.65
Urban	169 (41.1)	36.35–46.06
Education level		
No education	150 (36.5)	31.87–41.38
Primary school	171 (41.6)	36.82–46.55
Secondary school	85 (20.6)	16.93–24.99
Graduated	3 (0.7)	0.19–2.30
College	2 (0.4)	0.08–1.94
Employment status		
Yes	217 (52.8)	47.85–57.70
No	165 (40.1)	35.40–45.08
Sick/disabled	27 (6.5)	4.45–9.53
Retired	2 (0.4)	0.08–1.94
Housing status		
Owner	189 (45.9)	41.11–50.94
Tenant	128 (31.1)	26.74–35.90
Living with family/friends	94 (22.8)	18.96–27.30
Ability to meet their basic needs		
Always	37 (9.0)	6.50–12.30
Most of the time	99 (24.0)	20.09–28.58
Sometimes	160 (38.9)	34.22–43.85
Never	115 (27.9)	23.74–32.64
Time taken to get to hospital		
< 1 h	263 (63.9)	59.12–68.60
1 h–2 h	75 (18.2)	14.70–22.40
> 2 h	73 (17.7)	14.26–21.48

**Table 3:** Clinical characteristics of the patients

Variables	N = 411 (%)	95 CI %
Duration on ART		
0–4	179 (43.5)	38.72–48.51
5–9	141 (34.3)	29.76–39.15
≥ 10	91 (22.1)	18.28–26.53
Adherence		
Excellent	337 (82)	77.86–85.52
Very good	30 (7.3)	5.06–10.37
Good	26 (6.3)	4.25–9.25
Acceptable	4 (0.9)	0.31–2.65
Poor	14 (3.4)	1.95–5.79
ART regimen		
1st line	321 (78.1)	73.73–81.94
2nd line	90 (21.9)	18.06–26.27

**Table 4:** Bivariate association of demographic, socioeconomic and clinical factors with poor adherence to ART

Variables		Odds ratio (95% CI)	p value
Gender	Female	1.39 (0.48–3.98)	0.53
Age (years)	< 40	6.32 (2.04–10.58)	< 0.01
Area of residence	Rural	2.53 (0.81–7.83)	0.14
Education level	No schooling	1.11 (0.42–2.93)	0.82
Employment status	Unemployed	1.80 (0.68–4.74)	0.22
Housing status	Owner	1.89 (0.72–4.99)	0.18
Ability to meet basic needs	No	2.70 (1.04–7.00)	0.03
Time taken to get to hospital	≥ 2 h	2.43 (0.88–6.71)	0.07
Years on ART	< 5	2.10 (0.79–5.54)	0.12
ART regimen line	1st line	2.30 (0.52–10.23)	0.38

^*^Category in parentheses represents the reference group

**Table 5:** Multivariate analysis of demographic, socioeconomic and clinical factors associated with poor adherence to ART

Variables		Odds ratio (95% CI)	p value
Gender	Female	1.76 (0.51–6.12)	0.37
Age (years)	< 40	7.92 (2.21–28.4)	0.001
Area of residence	Rural	0.65 (0.19–2.27)	0.5
Education level	No schooling	0.99 (0.34–2.94)	0.99
Employment status	Unemployed	0.72 (0.21–2.46)	0.6
Housing status	Owner	0.35 (0.12–1)	0.05
Ability to meet basic needs	No	0.36 (0.11–1.15)	0.08
Time taken to get to hospital	≥ 2 h	0.61 (0.19–1.95)	0.4
Years on ART	< 5	0.79 (0.27–2.32)	0.67
ART regimen line	1st line	0.57 (0.12–2.79)	0.49

### Ethics

The protocol of this study was approved by ZL Institutional Review Board and the medical board of St Therese Hospital and written informed consent was obtained from all study participants.

## Results

The sample (*n* = 411) represented approximately 40% of the active patients of the HIV/TB clinic at St Therese hospital. The majority of the population was female (65%) and the mean age was 43.7 years. Nearly 38% were under the age of 40 years. The highest percentage of participants, 27.4%, were between 40–49 years of age.

Participants came mostly from rural areas (58%). While most completed primary school (41%), less than 2% graduated from high school or attended college. Forty percent were unemployed and the majority reported owning their own house (45%). Only a few participants indicated that they were always able to meet their basics needs (9%). Approximately 36% took less than 1 h to travel to the clinic.

PLH in our study with under 5 years of ART represented 43% of the sample, while 34% had between 5–9 years of ART and 22% had 10 years or more. The mean time receiving ART was 5.8 years ±12.49. A vast majority (78%) were on the 1st line regimen.

Most patients, 82%, reported that their adherence to ART was excellent. A subgroup analysis was performed, comparing groups on 1st and 2nd line treatment, defined based on our national guidelines on ART regimens [20]. There was no difference in adherence between patients on 1st line versus 2nd line treatment.

Age and ability to meet basic needs were two factors with a significant relationship with poor adherence: the odds of having poor adherence was significantly higher in patients under 40 years compared to those 40 years of age and older (OR: 6.32, 95% CI 2.04–10.58, p < 0.01). Patients who could not meet their basic needs were more likely to have poor adherence (OR: 2:70, 95% CI 1.04–7.00, p = 0.03). There was also no difference in the adherence based on gender, area of residence, time taken to get to the hospital, years on ART, education level, employment status and housing status.

## Discussion

To our knowledge, this was the first study to assess ART adherence among PLH in the central region of Haiti. Based on their self-descriptions, the majority of the PLH were females, under 50 years of age, living in a rural area within 1 h travel from the hospital. Most were homeowners, received at least primary school education, had a job, and were “sometimes” able to meet their basic needs. Most received ART for 10 years, were on the first line ART regimen based on national guidelines, and estimated that their adherence was “excellent”. Only two factors showed a significant association with poor adherence: age and their ability to meet their basic needs in bivariate analysis, with the latter demonstrating marginal significance in multivariate analysis.

Age was found to be an important factor related to poor adherence in our study. Patients under the age of 40 were more likely to have poor adherence than patients over 40 years of age. Our results are consistent with previous studies in similar settings [20, 21]. According to these studies, older patients had better compliance with their medication regimen because they were more used to the routine. The survival instinct was also mentioned in these studies: the elderly patient, in decline, recognized that their life expectancy would be prolonged through good adherence. Adolescents and young adults were at increased risk of treatment failure due to multiple social, psychological and adherence barriers [22].

The number of years receiving treatment was not associated with poor adherence according to our data. According to Kayser et al. a diagnosis of HIV of 5 years or greater was associated with a poorer adherence [23]. It is possible that patients who were on therapy for a longer duration may be more used to taking their medication, are likely to be more stable with respect to their treatment and disease management, and hence may be more likely to demonstrate better adherence. Patients who were on therapy for a longer period may also be more aware of the risks of non-adherence with respect to prior experience of the emergence of disease symptoms, e.g.: history of opportunistic infections and co-morbidities. As for the other clinical characteristics that we assessed in this study, we did not find any studies that investigated the relationship between the therapeutic regimen and compliance, although some studies found poor compliance in patients experiencing side effects to ART [8, 12].

PLH’s self-reported inability to meet their basic needs was the only variable apart from age to show a statistically significant relationship with adherence in bivariate analysis. Although this variable was marginally significant in multivariate analysis, this may be due to limited statistical power. Interestingly, housing status was also marginally significant in the multivariate analysis, which can also be linked with challenges in meeting basic needs. This is in spite of the fact that ART is free of charge in Haiti; were treatment not free, it would likely pose an even greater barrier to treatment adherence. This finding might be possible if they have given up hope of getting better with medication. If they do not have enough resources for food, they are likely to be more focused on solving this immediate need, instead of achieving viral load suppression. It is also a common belief amongst Haitians that medication should not be taken on empty stomach. Living in poverty, not having access to water/food may prevent from taking their medication. Only 9% said they could always meet their basic needs compared with 27.9% who could never meet their needs. Not being able to meet their basic needs was associated almost a three times greater odds of poor adherence.

There was no significant relationship with adherence and several other variables that could have potentially been linked to a patient’s ability to support him/herself. There was no difference in adherence by employment status. However, another study found that the direct effects of workload could interfere with patient adherence due to physical and mental fatigue, work schedule, etc. [24]. Being a non-homeowner, according to a study in the United States, was associated with poor adherence [8]. More than half of our sample either rented or lived with family or friends. Living in poor housing conditions could have contributed to poor adherence due to the patient having been concerned about his/her living conditions. The degree of social interaction, however, was also found to be associated with adherence whereby living alone has been shown to be associated with poor adherence [8] while interacting with people and social activities was shown to have a positive effect on adherence [25].

Education was another area where we did not find a clear association with adherence. Haiti is a country where the literacy rate for adults (15 years and over) is only 61% [7], and the Central Plateau region of the country has the highest rate of illiteracy. Accordingly, the level of education of the sample was generally very low. Other studies demonstrated a link between HIV morbidity and mortality with the level of educational attainment, even in a setting with more affluent socioeconomic context. Surprisingly, we found no statistically significant difference between adherence among those that had been schooled compared with those who had received no schooling. Other studies demonstrated a link between HIV morbidity and mortality with the level of educational attainment, even in a setting with a higher average level of overall educational attainment than ours. One study reported a positive association between education level and adherence [26]. It is possible that limited variability of literacy and educational attainment might have resulted in no association with adherence for these factors in the present study; however, given the high rates of illiteracy and the limited ability to meet basic needs it is likely that access to education could potentially improve adherence to ART in this area.

In this study, PLHs’ challenges with “meeting their needs” may have served as a general indicator of the poor socioeconomic conditions of the study participants. Low income was associated with poor adherence in some studies [27, 28]. Conversely, income level was also not associated with poor adherence in a study in India, where ART was free as it was in this study.

Lengthy travel time could pose a problem for ART refills and adherence with visits. Most participants lived in rural regions (58.9%). A previous study revealed poorer adherence amongst patients in rural areas compared with those from urban areas [29]. Among the obstacles reported in that study were difficult and expensive means of transport. The roads in Central Haiti are, overwhelmingly, in poor condition. The means of transports are limited to taxi-motorcycles with no bus or taxi-vehicles travelling between Hinche and neighboring cities. Moreover, in the rainy season, it is even more difficult to access the city where the hospital is located. A study carried out in a socio-economic context which was similar to ours also confirmed that distance plays a significant factor in patient compliance [30]. In yet another study, not having money for transportation was a statistically significant factor in poor adherence [31].

This study noted no difference in adherence by gender, despite the greater overall burden of HIV among women in Haiti. According to the Haiti Demographic and Health Survey VI (EMMUS VI), 2.3% of women are infected with HIV compared to 1.6% of men. In the Central Department, 1.5% of women are infected compared to 0.9% in men [32]. One study reported a lower number of ART clinic visits among women compared with men. However, assistance with childcare was associated with a notable increase in clinic attendance [33]. In light of these previous findings, we’ve been led to hypothesize that gender would have been associated with poor ART adherence, but our data noted no difference.

The model is overall well specified with a relatively low pseudo R2 (0.1142), possibly due to the fact that there are few predictors in the model. However, the likelihood ratio is statistically significant (p = 0.0019), thus proving that the predictors, although two in number, are adequate. In addition, the log likelihood does not deviate too much from 0, (Log likehood = − 66.167). So while a model with more predictors would have been more appropriate, we cannot question the overall specification of our model. Unlike the results of the bivariate analysis, the multivariate analysis reveals that only one factor is statistically significant associated with compliance: the age of the patients (p = 0.002, 0dd ratio = 6.16, 95% [1.97–19.21]). A patient aged 40 and over sees his chances of having an adherence increase 6 times compared to a patient under 40 years old. Several explanations are possible: the ability to meet one's needs can be a confounding factor, if it is also related to age. It is possible that this is also an explanatory factor with the same effects as age.

There were a number of limitations to our study. First, the proportion of patients who described having “poor adherence” was very small which limited our analysis. Second, adherence was estimated by patients self-reports, using a clinically accepted but subjective method. Thus, there could have been the risk of compromised patient recall and/or socially desirable responses which, could have potentially reduced the validity of the measure. Third, the study did not include pill counts. Therefore, we did not have an objective measure of the adherence of participants. Fourth, we did not assess clinical parameters such as clinical stage, medical history, presence of opportunistic infections, CD4 count and viral load. These factors could have directly impacted, and have been impacted by patient adherence. Fifth, this study was based only in Central Haiti and may not be generalizable to the rest of Haiti, nor to other settings. Sixth, the cross-sectional nature of the study meant that only associations were assessed, and causal inferences could not be made. The study may have been underpowered to detect significant associations for some of the variable studied.

## Conclusion

This study represents a crucial first step in identifying potentially high risk groups of PLHs who may need help to improve their adherence to ART therapy, based on socio-demographic and economic determinants of adherence to HIV treatment. Among the variables tested, younger age and inability to meet one's basic needs were both factors related to self-reported poor adherence. Particular attention should also be paid to the distance and/or mode of transport of patients to healthcare facilities, especially those in rural areas and also to those that are earlier on in their disease management journey who may need more help with their treatment. Further studies in other HIV patient care centers, using more objective methods of adherence measurement and exploration of other potential predictors of adherence are recommended.

## Data Availability

The datasets used and analyzed during the current study are available from the corresponding authors through reasonable request.

## Abbreviations

ART: Antiretroviral therapy
CI: Confidence interval
HIV: Human immunodeficiency virus
OR: Odds ratio
PLH: People living with HIV
TB: Tuberculosis
WHO: World Health Organization
